# Are Office-Based Workplace Interventions Designed to Reduce Sitting Time Cost-Effective Primary Prevention Measures for Cardiovascular Disease? A Systematic Review and Modelled Economic Evaluation

**DOI:** 10.3390/ijerph16050834

**Published:** 2019-03-07

**Authors:** Lan Gao, Phuong Nguyen, David Dunstan, Marjory Moodie

**Affiliations:** 1 Deakin Health Economics, Centre for Population Health Research, Deakin University, Geelong, VIC 3127, Australia; phuong.nguyen@deakin.edu.au (P.N.); marj.moodie@deakin.edu.au (M.M.); 2 Global Obesity Centre, Centre for Population Health Research, Deakin University, Geelong, VIC 3127, Australia; 3 School of Biomedical Sciences and Pharmacy, The University of Newcastle, Callaghan NSW 2308, Australia; 4 Baker Heart and Diabetes Institute, Melbourne 3004, Australia; David.Dunstan@baker.edu.au; 5 School of Public Health, The University of Queensland, Brisbane 4072, Australia; 6 School of Sport Science, Exercise & Health, University of Western Australia, Perth 6907, Australia; 7 School of Exercise and Nutrition Sciences, Deakin University, Melbourne 3125, Australia; 8 School of Public Health & Preventive Medicine, Monash University, Melbourne 3800, Australia; 9 Mary MacKillop Institute for Health Research, Australian Catholic University, Melbourne, VIC 3003, Australia

**Keywords:** sedentary behaviour, workplace intervention, multicomponent, cost-effective analysis, cardiovascular disease, primary prevention

## Abstract

Objectives: To assess the cost-effectiveness of workplace-delivered interventions designed to reduce sitting time as primary prevention measures for cardiovascular disease (CVD) in Australia. Methods: A Markov model was developed to simulate the lifetime cost-effectiveness of a workplace intervention for the primary prevention of CVD amongst office-based workers. An updated systematic review and a meta-analysis of workplace interventions that aim to reduce sitting time was conducted to inform the intervention effect. The primary outcome was workplace standing time. An incremental cost-effectiveness ratio (ICER) was calculated for this intervention measured against current practice. Costs (in Australia dollars) and benefits were discounted at 3% annually. Both deterministic (DSA) and probabilistic (PSA) sensitivity analyses were performed. Results: The updated systematic review identified only one new study. Only the multicomponent intervention that included a sit-and-stand workstation showed statistically significant changes in the standing time compared to the control. The intervention was associated with both higher costs ($6820 versus $6524) and benefits (23.28 versus 23.27, quality-adjusted life year, QALYs), generating an ICER of $43,825/QALY. The DSA showed that target age group for the intervention, relative risk of CVD relative to the control and intervention cost were the key determinants of the ICER. The base case results were within the range of the 95% confidence interval and the intervention had a 85.2% probability of being cost-effective. Conclusions: A workplace-delivered intervention in the office-based setting including a sit-and-stand desk component is a cost-effective strategy for the primary prevention of CVD. It offers a new option and location when considering interventions to target the growing CVD burden.

## 1. Introduction

Cardiovascular disease (CVD) has been the dominant cause of mortality in Australia for the past several decades, with coronary heart disease (CHD) and stroke ranking the highest among the leading causes of death [1,2].

CVD is considered largely preventable by modification of related risk factors, like smoking, obesity, physical inactivity, inadequate consumption of fruits and vegetables, high levels of blood glucose, blood pressure and lipids. Ninety percent of Australian adults have at least one modifiable risk factor for CVD, while 64% have three or more [3]. For instance, 90% of the risk associated with myocardial infarction (MI) worldwide is attributable to these risk factors [4]. Although mortality due to CVD is expected to decline over time given advancements in prevention, early diagnosis and treatment, the total CVD burden is estimated to increase over the next few decades given the ageing population[1].

Primary prevention offers the best option for tackling the growing prevalence of CVD worldwide. Australian and international primary care guidelines unanimously emphasise comprehensive risk assessment to enable effective management of identified modifiable risk factors through lifestyle changes and/or pharmacological therapy [5,6,7].

The link between excessive sitting time and adverse health outcomes, including CVD, type 2 diabetes and premature mortality, has been well recognised even after accounting for the influence of moderate to vigorous physical activity [8,9]. Persons who sit 8 to 11 hours per day have an estimated 15% increased risk of death over the next three years compared to those who sit less than 4 hours per day [10]. Too much sitting has become a key public health concern [11], since sedentary behaviour occupies more than half of adults’ waking hours [12]. In office workers, workplace sitting is the single biggest contributor to daily sitting time [13]. Further, as office-based workers constitute the largest occupational sector and the proportion of industrial work that involves sedentary activity is increasing [14], the office workplace has been identified as a key setting in which to reduce prolonged sitting time [15].

In contrast to the detrimental effects of prolonged sitting, large scale prospective observational studies have reported that time spent standing is linked to a reduction in all-cause mortality risk in a dose-response manner [16,17]. Furthermore, analyses using isotemporal substitution modelling have shown that replacing sitting time with equal amounts of standing is linked to substantial mortality risk reduction in Australian adults [18]. In the workplace context, a reduction in time spent sitting results in increased physical activity that is usually of light intensity, including increased standing. Recent workplace intervention trials in Australia and the UK using device-based measures have reported that the ~42 minute reduction seen in workplace sitting at 12 months is almost entirely achieved by an equivalent increase in standing [19,20]. In the Australian study, the intervention resulted in a small benefit for fasting glucose and the overall cardiometabolic risk score at 12 months [21]. Collectively, the observational and intervention evidence suggests that workplace approaches leading to modest reductions in sitting time through increased standing may have some benefit for CVD prevention in the long-term.

To date, none of the studies evaluating interventions that reduce workplace sitting time and increase standing time report participant outcomes in the period after the end of the intervention due primarily to insufficient length of follow-up. Therefore, the effectiveness of interventions that decrease workplace sitting time in reducing the prevalence of CVD events and their associated long-term cost-effectiveness credentials remain unknown.

The current study aimed to (i) systematically identify workplace-delivered interventions targeted at reducing sitting time of office-based workers leading to increased standing; (ii) simulate the long-term CVD outcomes associated with these interventions; and (iii) assess their cost-effectiveness.

## 2. Methods

### 2.1. Systematic Review of Evidence

#### Search Strategy

To the best of our knowledge, there has been only one systematic review of workplace interventions for reducing sitting time in office workplaces [22], their search was conducted on 9 August 2017. An updated literature search was undertaken to identify any new studies published thereafter in Medline (plus PsyInfo via EBSCO) and Embase (see Appendix for search strategy). The search was carried out on 10 July 2018 using the same search terms and inclusion and exclusion criteria as reported in the systematic review by Shrestha [22]. Briefly, workplace interventions usually comprised of multiple components such as sit-stand desk, counselling, active workstation (i.e., desks that can substantially promote energy expenditure compared to sit-desks), information/feedback and/or reminder, computer prompts, mindfulness training, activity tracker, organisational support. In the study by Shrestha, only those multi-component interventions involving a sit-and-stand desk were able to significantly reduce the workplace sitting time with most of the evidence being of low quality due to the limitation in study protocols and small sample sizes [22]. Risk of bias was assessed using Cochrane Risk of bias tool [23].

If more than one study was identified, the effect size was meta-analysed in Review Management (RevMan) 5.1 (Copenhagen: The Nordic Cochrane Centre, The Cochrane Collaboration, 2014, London, UK) using minutes of standing time (per eight hours of working time), incorporating the inverse variance method with random effects model. Trials with unadjusted estimates of intervention effects were calculated from the raw data provided in published papers. Heterogeneity was assessed using the I^2^ statistic.

### 2.2. Modelling the Long-Term CVD Outcome

#### 2.2.1. Population

Office-based workers in Australia aged between 30 and 65 years and without prior history of CVD were modelled. The modelled population was divided into 5-year age and gender groups based on Australia Bureau of Statistics (ABS) data [24], with average weight of each group derived from the 2014-15 Australia National Health Survey [25].

#### 2.2.2. Model Structure

A pre-existing Markov model consisting of seven health states (healthy, post-coronary heart disease (CHD), post-stroke, death due to incident CHD, death due to incident stroke, death post-CHD, death post-stroke and death due to all other causes) was used [26,27,28] (Figure S1). Only CHD and stroke were modelled since these two events account for the majority of total CVD events [29]. Ischemic and haemorrhagic stroke were not separately simulated, however, the difference in case fatality of these two types of stroke was taken into consideration. All persons in the hypothetical cohort started from a healthy state. In each Markov cycle, healthy subjects may stay healthy, have a CHD or stroke event (fatal or non-fatal) or die from other non-CVD causes. For subjects who experience CVD, they either survive or die from the event. CVD survivors transit to the post-CHD or post-stroke health state corresponding to the CVD event that occurred in the previous cycle. To simplify the model and given our primary prevention focus, only an individual’s first CVD event was simulated over their lifetime. The modelling was performed using TreeAge (TreeAge Pro 2018, R2.1. TreeAge Software, Williamstown, MA, USA).

#### 2.2.3. Outcome Measures

Increases in standing time (min/week) was selected as the intervention effect, given that reduction in sitting time is mostly replaced by standing time. Studies that have used objective measures of sitting, standing and stepping allow ascertainment of the activities replacing sitting time) and most studies report this outcome. Increase in time spent standing was multiplied by the metabolic equivalent units (MET) associated with standing and then converted to energy expenditure using the following equation [30]

(1) Energy expenditure (Kcal/min)=MET×3.5×Body weight (kg)200

Given insufficient evidence of decay effects, it was hypothesised that the increase in standing time was sustained until 5 years post intervention, which is consistent with a previous modelled economic study around physical activity interventions [31].

#### 2.2.4. Disease Inputs

The baseline incidence (The 28 day survival incidence was defined as the number of people who survive the first 28-days after a first ever CHD or stroke event divided by the total number of population in that age and gender group) of CHD and stroke and mortality from all other causes were extracted from the Australian Burden of Disease study [32]. The 28-day and post-28 day case fatality rates of CHD and stroke were derived from previous studies of primary prevention interventions for CVD in Australia [26,27]. The probabilities of non-fatal CVD events in the first year were computed using CVD incidence, post-28 day case fatality and CVD incidence trends [26]. The incidence and case fatality of CHD and stroke were assumed to decline over time at a rate of 2.0% annually [28], given advancements in disease prevention and management. Mortality due to all other causes was assumed to decrease at a rate less than incidence and case fatality [28]. It was assumed that the changes in disease and mortality trends were maintained for 10 years only and thereafter remained constant.

Studies have shown that physical activity as measured by energy expenditure is associated with reduced occurrence of CVD events in healthy populations [33]. Therefore, in order to adjust for the reduced incidence of these CVD events in the intervention group, the relative risks (RR) of CHD and stroke corresponding to different intensities of energy expenditure between the intervention and control groups were applied.

#### 2.2.5. Costs

An Australian healthcare system perspective was adopted for the measurement of costs. Productivity gains or losses associated with the intervention were excluded. Costs of each of the workplace-delivered interventions were assigned as one-off costs and based on published literature where applicable. The unit costs of treating an incident case of CHD and stroke were sourced from the Independent Hospital Pricing Agency (IHPA), Australia, while management costs attributable to ongoing CHD and stroke care were derived from published literature. All costs were expressed in Australian dollars (AUD) for the 2016 reference year (Appendix Table A1).

#### 2.2.6. Utility Weights

A utility weight was attached to the time spent in each health state to capture the average quality of life experienced by each age and gender group; quality-adjusted life years (QALY) were accrued over the entire time horizon of the model (Appendix Table A1). Separate utility weights were derived from the published literature for patients with CHD and stroke.

#### 2.2.7. Cost-Effectiveness Analysis 

Using the Markov model prediction of QALYs lived and costs over the cohort’s lifetime for both the intervention and control group, an incremental cost-effectiveness ratio (ICER) was calculated, with future costs and benefits discounted at 3% annually [34]. One-way sensitivity analyses were conducted to test the robustness of the base-case results. Where applicable, key model parameters were varied within a particular range (based on the best available evidence or a 20% increase or decrease in costs where data were insufficient to inform the range). One-way sensitivity analyses results are shown in a Tornado diagram, which graphs sequentially the variables with the largest impact on the cost-utility results.

Probabilistic sensitivity analyses (PSA) were performed to assess the overall impact of uncertainty in the model by defining distributions of key model parameters (i.e., transition probabilities, utility weights and costs). Five thousand iterations (i.e., second-order Monte Carlo simulations) were run to obtain a mean and 95% confidence interval for the corresponding costs and benefits; the results were plotted on a cost-effectiveness plane. The often cited willingness-to-pay (WTP) per QALY of AUD50,000 [35] was adopted to determine the cost-effectiveness of the individual workplace-delivered interventions, with an acceptability curve generated for each interventions.

If the intervention was implemented nationally, it was conservatively assumed that 20% of the entire Australian workforce (45%) who work in a sedentary occupation would participate [36].

#### 2.2.8. Ethical Statement

Since all the data used to inform the health economic model were sourced from published literature, the ethics approval was not required.

## 3. Results

### 3.1. Systematic Review of Workplace Interventions to Reduce Sitting Time

The most recent systematic review of workplace interventions targeting sedentary activity compared the effectiveness of different interventions [22] which included one or more of the following components:

- Physical changes in workplace design and environment including changes in desks, chairs and workplace layout;
- Policies to modify the organisation of work including arranging walking meetings, encouraging breaks and completing sitting diaries;
- Provision of information and counselling including signs or prompts at the workplace, e-health interventions, distribution of leaflets and counselling.

In addition to the above inclusion criteria, our study stipulated that for interventions involving a sit-and-stand desk, each participant was allocated a sit-and-stand desk for use over the entire duration of their work hours (rather than occasional use subject to availability).

**Figure A1:**
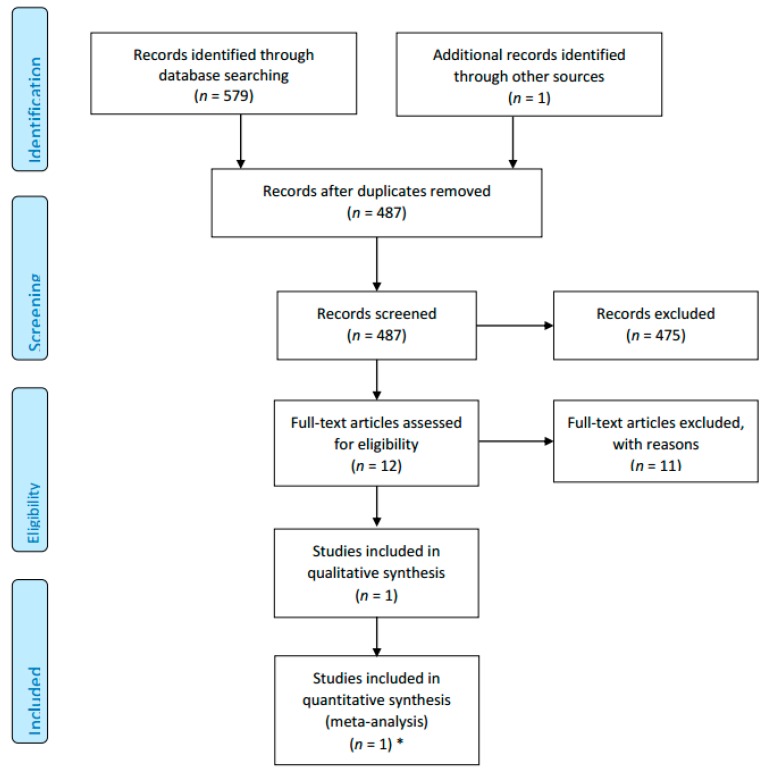
PRISMA Flow Chart. * [37].

Our updated literature search identified 579 additional articles published between August 2017 and July 2018. After screening titles and abstracts, 475 articles were excluded due to irrelevance. Eleven articles were reviewed on a full-text basis, resulting in nine being excluded due to ineligible study design (*N* = 5), wrong outcome (*N* = 2) and different setting of interest (*N* = 2). Eventually, one new study was included in our systematic review [19,37,38] (the previous systematic review included 19 studies in the meta-analysis [22]) (see Appendix Figure A1).

The newly identified study, Zhu 2018 [37] was a cluster randomised controlled trial (cRCT) which enrolled participants from the United States. Work sites were randomised into intervention or control; office-based workers in the intervention arm received a multi-component intervention involving a sit-and-stand workstation during both an active (4 months) and a maintenance phase (14 months). The characteristics and risk of bias assessment of the additional study are summarised in Appendix Table A2.

Given that the sample size of studies included in the original systematic review (Analysis 1.6) ranged from 16 to 44 [22] and only the short-term outcomes (i.e., month 3) were meta-analysed, it was considered inappropriate to meta-analyse them with the addition of the newly identified study. Another reason was the heterogeneity in study design; the sit-and-stand desk was not necessarily assigned to each trial participant on a one-to-one basis (e.g., sit-and-stand desk provided in common area or only for the first 3 months), Hence, it was decided in the base case analysis of the current study, for the intervention involving a sit-and-stand desk, that the intervention effect size would be based on meta-analysis of two studies only (Healy 2016 plus the newly identified study Zhu 2018) at month 12, whilst in the sensitivity analysis, the results from the study with the largest sample size (i.e., Healy 2016) were used (Figure S2).

For all the other interventions, the model inputs in terms of the changes in standing time are summarised in Table 1.

### 3.2. Results of Modelling

#### 3.2.1. Cost-Effectiveness Analysis

Implementation of the intervention involving a sit-and-stand workstation component was associated with both higher benefits (23.280 QALYs versus 23.273 QALYs) and costs ($6820 versus $6524). The resultant ICER was $43,825 per QALY gained, which makes it cost-effective. If the intervention was scaled up to 20% of the national office-based workforce, it would result in a total gain of 4335 QALYs for an additional total cost of $267M (the cost offset due to avoided CVD was $83M). Specifically, it could potentially avoid 70 incident non-fatal CHD and 20 incident fatal CHD events per 100,000 population whereas no difference in terms of fatal or non-fatal event of stroke (results generated from the economic model).

#### 3.2.2. Sensitivity Analysis

**Figure 1:**
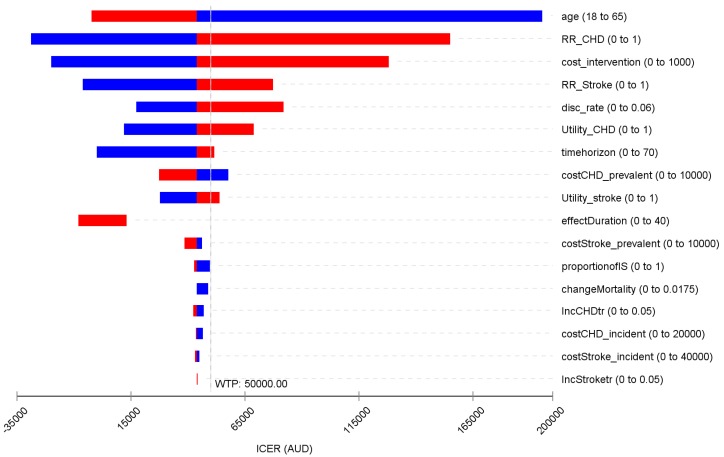
Tornado diagram for the deterministic sensitivity analyses. WTP: willingness to pay; ICER: incremental cost-effectiveness ratio; AUD: Australia dollar; CHD: coronary heart disease; RR: relative risk. Note: blue bar means the ICER decreases as parameter value increases; red bar represents ICER increases as parameter value increases.

The base case results were most sensitive to the target age group for the intervention, RR of CHD and stroke relative to the control, the intervention cost and discount rate (Figure 1). When targeted at an older age group, the intervention became more cost-effective (and vice versa). Reduction in incidence of CHD was a key determinant of the ICER; the threshold RR of intervention versus control in incidence of CHD was 0.972.

**Figure 2:**
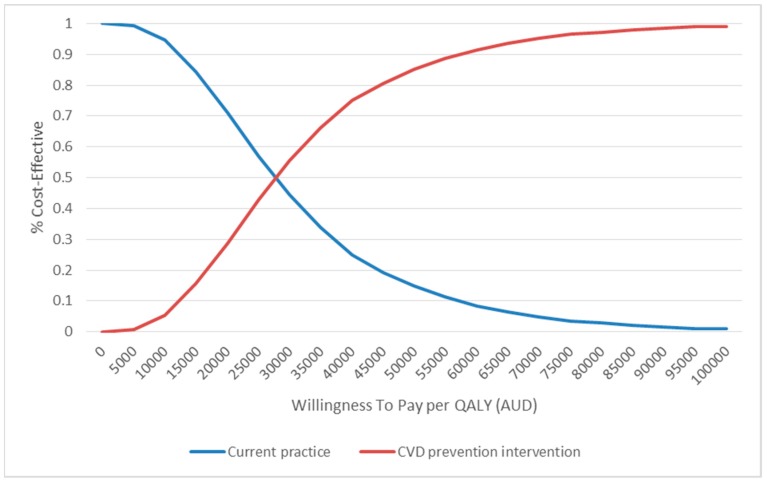
Cost effectiveness acceptability curve. QALY: quality-adjusted life year; Note: the y-axis represents the probability of the intervention being cost-effective as determined by the incremental cost-effectiveness ratio; the x-axis represents the change in WTP/QALY threshold.

**Figure 3:**
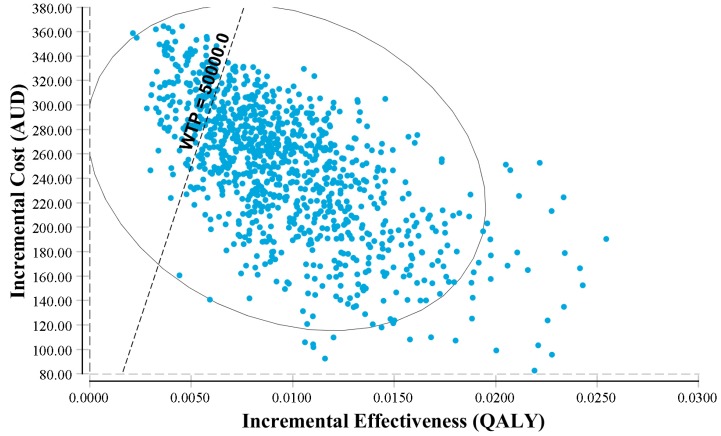
Incremental cost-effectiveness plane. Note: QALY: quality-adjusted life year; WTP: willingness to pay; AUD: Australia dollar. The y-axis represents the incremental costs of proposed (with the workplace intervention for CVD prevention) vs. current scenarios (without the workplace intervention for CVD prevention); the x-axis presents the incremental QALY gains of the proposed vs. current scenarios. The workplace intervention has a probability of 85% being cost-effective.

The PSA yielded similar results to the base case scenario, with the point estimate of the base case falling well within the 95% CI (Table 2). The cost-effectiveness acceptability curve showed that if the WTP/QALY was above $35,000, the intervention had a >66% probability of being cost-effective (Figure 2). The cost-effectiveness plane echoed these results, suggesting a significantly high probability of being cost-effective (Figure 3).

## 4. Discussion

Our study systemically reviewed the most recent evidence around the efficacy of workplace delivered interventions, which aimed to reduce office-based workers’ sitting time leading to increased standing and undertook a modelled economic evaluation to simulate the long-term health benefits of such interventions in preventing CVD in Australia. One new trial was identified which informed the effect size (i.e., increase in standing time) in the modelled cost-effectiveness analysis of workplace intervention. This intervention was highly likely (>85%) to be cost-effective for primary prevention of CVD in the Australian context.

Increasing evidence shows that excessive sedentary time is related to increased all-cause and cardiovascular morbidity and mortality [39]. Preventative efforts have targeted sedentary behaviour. Whilst national sedentary behaviour guidelines for Australia [40], Canada [41] and United Kingdom [42] recommend that adults should minimise time spent being sedentary for extended periods, no prescriptive recommendation has been provided about the maximum limit of sedentary time. Recent meta-analyses and reviews have assessed the prospective evidence on the association of sedentary behaviour with CVD outcomes [39,43]. These reviews have consistently suggested that excessive sedentary behaviour is associated with increased incidence of CVD (pooled RR 2.47, 95% CI 1.44–4.24) [43] and higher CVD mortality (pooled HR 1.90, 95% CI 1.36–2.66) [43,44]. The American Heart Association summarised the current evidence surrounding sedentary behaviour as a potential risk factor for CVD [39]. Therefore, in order to provide more evidence to underpin the current national sedentary behaviour guidelines, it is important to explore the quantitative association between reduction in sitting time and long-term changes in CVD.

The current recommendation for primary prevention for CVD relates to assessment of the risk factors [6]. For all persons with CVD risk factors, the first-line treatment is lifestyle behaviour-change, which typically incorporates behavioural change counselling. However, it was reported that the current recommendation for lifestyle behaviour-change interventions should be revisited due to their poor value for money given marginal population health gains and a very high cost [26]. If an individual’s CVD risk factors are above the level set for treatment, lipid-lowering, antihypertensive or antiplatelet medication could be initiated, however treatment compliance is suboptimal, with an estimated 40% dropout rate in the first 12 months [45]. Hence, there is a need to investigate more primary prevention options for CVD, especially for people of working age. Workplaces offer the potential for generating intrinsic social support and are well-positioned for the delivery of interventions designed to promote population health. The active collaboration of workplace peers in making maintainable modifications to achieve a healthy lifestyle, might reduce the individual effort and motivation needed to make behavioural changes and result in a more sustainable long-term effects [46].

It is reported that a greater amount of sitting time together with prolonged sitting time was negatively associated with changes in high density cholesterol (HDL), triglycerides and 2-hour post load glucose [47], animal-based studies suggest that this deleterious effect might be contributed by the reduction in lipoprotein lipase activity [43] and upregulated insulin resistance [48]. However, we did not model the long-term health outcomes of reduced sedentary behaviour through a reduction in CVD biomarkers (i.e., LDL, insulin resistance etc.) as there is no consistency in these outcomes [49], whereas the increase in standing time is fairly consistent across studies.

To the best of our knowledge, our study is the first to assess the cost-effectiveness of interventions to reduce sitting time as a primary prevention measure for CVD. The intervention effect was mediated by increased standing time (i.e., reduction in sitting time was largely replaced by standing time), which contributed to lower incidence of CVD events. Prior modelling studies of the primary prevention of CVD adopted a similar model structure and showed that compared to current practice, medical treatments including antihypertensive (i.e., ACE inhibitor, Calcium channel blocker etc.), lipid lowering (statin) and antiplatelet (i.e., aspirin) drugs were normally cost-effective options when administered to people with increased CVD risk [26]. First-line therapy using lifestyle program or dietary advice targeted at similar populations was associated with an ICER much higher than $50,000/DALY, rendering them not cost-effective under an Australian healthcare system perspective [26]. Our intervention, on the contrary, targeted at a general population office-based workforce not necessarily with increased risk of CVD, yielded an ICER of $18,221/QALY, with a potential to avoid 230 incident non-fatal CHD events and 50 incident non-fatal strokes per 10,000 population in Australia. A 2014 review pointed out that replacing sedentary time with an equal amount of either sleep or light-intensity activity yielded similar reductions in CVD biomarkers (2.2% reduction versus 2.4% reduction in insulin respectively) [50], which suggests that the intervention effect of reducing sitting time is likely to be additive to other interventions including promoting physical activity.

In order to formulate an evidence basis for guideline-making, we also tested the impact of different reductions in sitting time (assumed 100% replaced by standing time) on long-term health outcomes. If sitting time could be lowered by two hours per day across entire working age groups, the intervention would become dominant (i.e., involve less cost and greater benefits). The number of total avoided CVD event would be 290 non-fatal CHD per 10,000 population whereas no difference in the number of non-fatal/fatal stroke (results generated from the economic model).

Our updated systematic review only identified one new study additional to the most recent published review of workplace interventions to reduce sitting time [22]. Only interventions involving a sit-and-stand desk with or without a counselling component were observed to be effective in reducing sitting time and increasing standing time, whereas other interventions entailing computer reminders or activity tracker did not result in significant between-group differences and therefore were not modelled [22].

When considering national implementation of a preventative intervention, affordability is a key concern. The cost of this multicomponent intervention is comparable to dietary advice and lifestyle program [26]. In addition, given that its potential benefits are partly received by employers (i.e., increased productivity and reduced presenteeism) [51], it is reasonable to suggest that the program’s implementation might be co-funded by employers to alleviate some of the burden on government.

This is the first study to model the impact of workplace interventions that has attempted to assess the impact of reduced sedentary behaviour on CVD primary prevention. It translates the short-term benefits observed during the trial (i.e., increase in standing time) into long-term clinical outcomes (i.e., avoided CHD and stroke). It provides preliminary evidence underpinning future recommendations around daily limits on sitting time. The key limitations of the study are, firstly, the uncertainty around the sustainability of the intervention effect; sensitivity analyses were run to test that. As suggested, intervention delivered in the workplace may be more maintainable than other non-workplace delivered interventions. Secondly, only the first CVD event was modelled and any subsequent events were omitted given the focus on primary prevention of CVD. Thirdly, the likely relationship between reduced sedentary behaviour and improved metabolic biomarkers (i.e., lipids) was not accounted for in the model given the lack of consensus about the association between sedentary behaviour and lipid level. Fourthly, it is worth noting that, given the potential link between prolonged standing and increased risk of vascular and musculoskeletal issues [52], we need to emphasise the importance of intermittently alternating standing with periods of sitting and physical activity to minimise such risks. Fifthly, the effect size of the workplace-delivered intervention was heavily weighted on the study by Healy [19], which is not uncommon when deriving an effect size from a meta-analysis. However, there is little evidence to suggest that Australian have attitudes towards adopting changes/new interventions that might be different to other cultures. With this generalisable intervention impact, one can populate the economic model with local costs and incidences of CVD to generate the country-specific ICER for country of interest within an international context. Lastly, caution needs to be applied when interpreting the results from this study. While reduced sedentary behaviour is linked to better CVD health [53], in the current modelled study, we have assumed that the change in sedentary behaviour is mediated by the increase in standing at workplace. However, it should be acknowledged that workplace interventions whereby reduced sitting is achieved increases in physical activity (stepping) are likely to be more favourable in relation to CVD prevention.

## 5. Conclusions

A workplace-delivered intervention employing a sit-and-stand desk component is a cost-effective option for primary prevention of CVD in the Australian context. The benefit was derived from reductions in time spent sitting that resulted in small increases in standing time in the workplace. This offers a new option and location in the campaign against the growing burden of CVD. The affordability of this intervention could be improved by partnerships with employers.

## Figures and Tables

**Table 1:** Meta-analysed results of reduction in standing time for different types of workplace interventions.

Intervention	Comparator	Changes in Standing Time (Min/Day, 95% CI)	*p*-Value
Sit-stand desk with or without information and counselling	Sit-desk	40.85 (26.18, 59.42) *	*p* < 0.001
Information, feedback and/or reminder	No intervention	10.24 (−17.17, 37.65)	*p* > 0.05
Prompts plus information	Information alone	32.40 (−6.81, 71.61)	*p* > 0.05
Computer prompts to step	Computer prompts to stand	−11.9 (−15.33, −8.47)	*p* > 0.05
Activity tracker combined with organisational support	Organisation support	3.40 (−19.80, 26.60)	*p* > 0.05

Footnote: the results except for the first row are sourced from the previous Cochrane systematic review [22]. * long-term results only.

**Table 2:** Results of cost-effectiveness analysis.

Groups	Base Case Results			
	Cost	QALY		ICER
**Intervention**	$6820	23.280		-
**Control**	$6524	23.273		-
Difference	$170	0.007		$43,825/QALY
**Probabilistic sensitivity analyses**				
	**QALY (mean, 95% CI)**		**Cost (mean, 95% CI)**	
**Intervention**	23.255 (23.104, 23.360)		$6342 ($5545, $7232)	
**Control**	23.245 (23.089, 23.354)		$6093 ($5288, $6998)	

QALY: quality adjusted life year; ICER: incremental cost-effectiveness ratio.

## Supplementary Materials

The following are available online at https://www.mdpi.com/1660-4601/16/5/834/s1, Figure S1: Illustration of Markov model structure, Figure S2: Forest plot of meta-analysed results.

## Appendix A

### Search Strategies

Our updated literature search initially identified 579 number of citations published between August 2017 and July 2018 (Embase: 425 hits; Medline complete and PsyInfo visa EbscoHost: 172 hits). After screening the titles and abstracts, 475 of articles were excluded due to irrelevance. Eleven of articles were reviewed on the full-text basis, among them, nine of papers were excluded due to the ineligible study design (*n* = 5), wrong outcome (*n* = 2), and different setting of interest (*n* = 2). Eventually, only one studies [37] were included into the quantitative analysis of the current study. The study selection process was illustrated in Figure A1.

### Search Strategies:

#### Embase

#1 sedentary
#2 ’sitting’/de
#3 ’seated posture’
#4 seated NEAR/1 posture
#5 chair:ab,ti OR desk:ab,ti
#6 chair:ab,ti
#7 desk:ab,ti
#8 office AND inactiv*
#9 #1 OR #2 OR #4 OR #6 OR #7 OR #8
#10 ’work’/de OR work
#11 work*
#12 ’occupation’/de OR occupation
#13 employe*
#14 #10 OR #12 OR #13
#15 effect
#16 control
#17 evaluat*
#18 intervention*
#19 program
#20 compare
#21 #15 OR #16 OR #17 OR #18 OR #19 OR #20
#22 #9 AND #14 AND #21
#23 #22 AND [embase]/lim
#24 #23 AND [humans]/lim AND [embase]/lim

#### Medline + PsyInfo (Via EBSCOhost Data Base)

#1 (work[tw] OR works*[tw] OR work’*[tw] OR worka*[tw] OR worke*[tw] OR workg*[tw] OR worki*[tw] OR workl*[tw] OR
workp*[tw] OR occupation*[tw] OR employe*[tw])
#2 (effect*[tw] OR control[tw] OR controls*[tw] OR controla*[tw] OR controle*[tw] OR controli*[tw] OR controll*[tw] OR evaluat*[tw] OR intervention*[tw] OR program*[tw] OR compare*[tw])
#3 (sedentary OR sitting) OR seated posture OR chair[tiab] OR desk[tiab] OR (office AND inactiv*)
#4 (animals [mh] NOT humans [mh])
#5 #1 AND #2 AND #3 NOT #4

**Table A1:** Model Parameters.

Parameters in the Model	Value Used in the Model	Source
Cost		
Intervention	$431	Gao et al., 2018 [51]
Incident stroke	$23,581	IHPA *
Prevalent stroke	$3201	Lim et al., 2005
Incident CHD	$17,863	IHPA *
Prevalent CHD	$4539	Lim et al., 2005
Utility weight		
CHD	0.86	Cobiac et al., 2012 [26]
Stroke	0.76	Cobiac et al., 2012 [26]

* National Hospital Cost Data Collection, Independent hospital Pricing Authority (IHPA) [54]. CHD: coronary heart disease.

**Table A2:** Characteristic of Included Study.

Zhu 2018		
Methods	Cluster-random allocation (quasi-experimental) Unblinded Study duration: 18 months Dropout: 0% (100% retention) **Location:** Two units of a university, located in different buildings in the US **Recruitment:** Employees in each worksite were invited to participate via email lists. Participants signed consent form.	
Participants	**Population:** full time office workers (18–65 years old) **Intervention group:** 24 participants **Control group:** 12 participants **Demographics:** Mean age: 39.1 (SD 11.3); female 70.8%.	
Interventions	**Duration of intervention:** 4 months **Intervention:** Each participants received a sit-stand workstation at their primary work location. Three additional treadmill workstations were installed in common areas. E-Newsletters for social support and maintaining progress were sent to all staffs. **Control:** Staffs received newsletter as in intervention arm but no environmental changes were made.	
Outcomes	**Outcome name, measurement time/tool (units of measurement)** Posture and activity were measured by accelerator. Outcomes reported in minutes/ day for each period of total sitting, total standing, total LPA, total MVPA, sit-to-stand transitions, time accrued in prolonged sitting. Cardio-metabolic biomarkers were evaluated by BMI. Blood pressure was taken twice in consistent time and manner for each participants. Full lipid profile with total cholesterol, high-density lipoprotein and low-density lipoprotein as well as high-sensitivity C-reactive protein, triglycerides, plasma glucose, and insulin levels were measured. Productivity loss was evaluated using questionnaires. Participants’ acceptance were evaluated by short interviews for intervention arm.	
Notes	This study was funded in part by the Virginia G. Piper Charitable Trust and the Steelcase Corporation. First author is supported by the Fundamental Research Funds for the Central Universities in China (GK201603128). Other authors are supported by the National Institute of Health (R01CA198971).	
*Risk of bias*		
**Bias**	**Authors’ judgement**	**Support for judgement**
Random sequence generation (selection bias)	High risk	Randomisation was not done as participants in intervention and control groups were selected from different location of one workplace.
Allocation concealment (selection bias)	High risk	Intervention and control groups were selected from two separate locations. However no information on allocation concealment
Blinding of participants and personnel (performance bias) All outcomes	Unclear risk	Not reported
Blinding of outcome assessment (detection bias) All outcomes	Unclear risk	Not reported
Incomplete outcome data (attrition bias) All outcomes	Low risk	Virtually no attrition
Selective reporting (reporting bias)	Low risk	All outcomes mentioned in the method section were reported. Study protocol was not available.
Baseline comparability/imbalance	Low risk	No significant difference between groups at baseline was detected.
Validity of outcome measure	Low risk	All questionaries used were validated and relevant to China context. But physical activity and sedentary time were measured by accelerators.
