# Supplementary figures and images for: Are Office-Based Workplace Interventions Designed to Reduce Sitting Time Cost-Effective Primary Prevention Measures for Cardiovascular Disease? A Systematic Review and Modelled Economic Evaluation
Source: Int J Environ Res Public Health. 2019 Mar 7;16(5):834. doi: 10.3390/ijerph16050834 (PMC6427179; doi:10.3390/ijerph16050834)

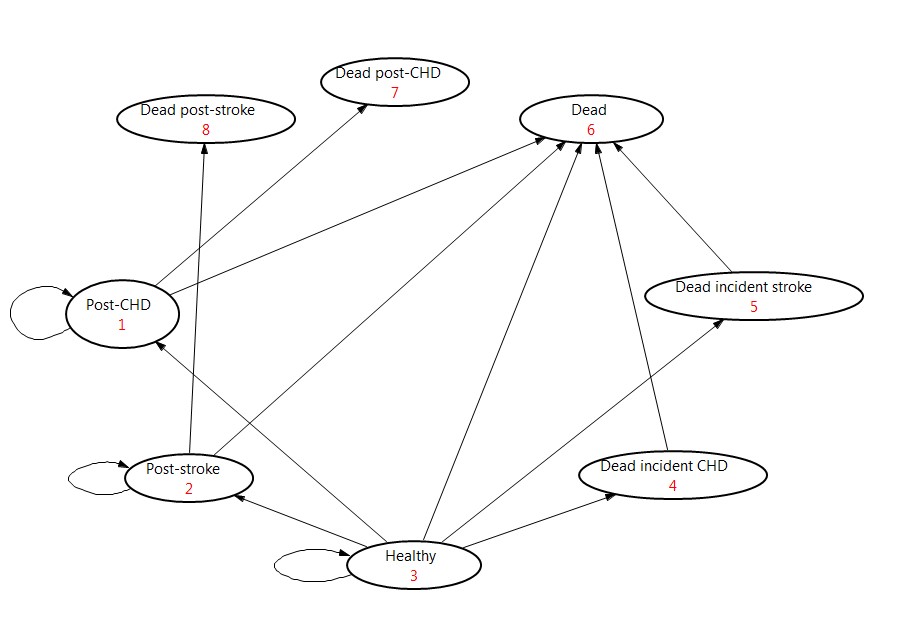


**Figure S1.** Illustration of Markov model structure.


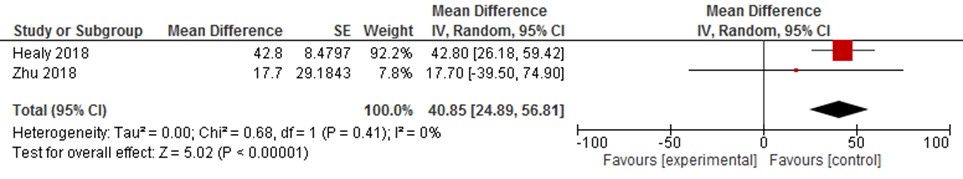


**Figure S2.** Forest plot of meta-analysed results.

Supplement: Supplementary file 1 [file ijerph-16-00834-s001.zip › ijerph-432792-suppl xml.docx]

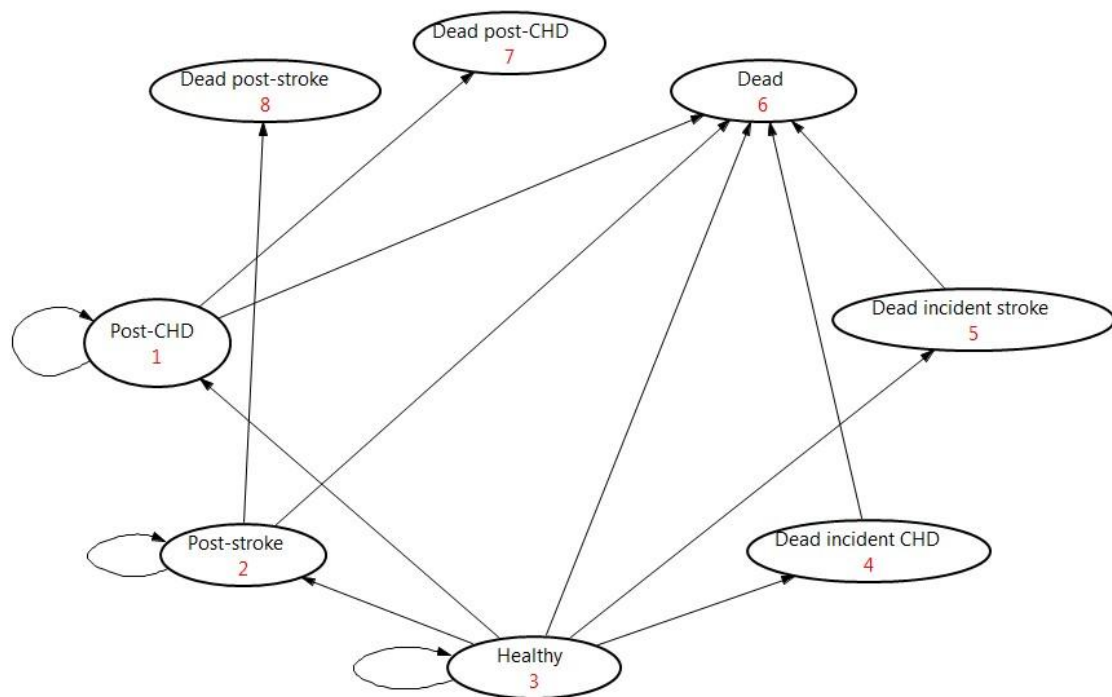

**Figure S1.** Illustration of Markov model structure.

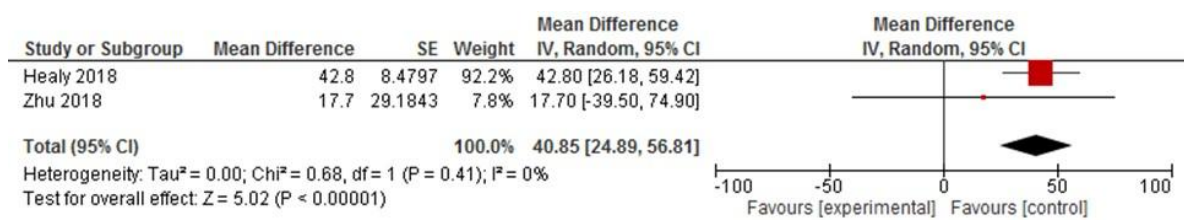

**Figure S2.** Forest plot of meta-analysed results.

Supplement: Supplementary file 1 [file ijerph-16-00834-s001.zip › ijerph-432792-suppl xml.pdf]
